# Synthesis of Epoxy Methacrylate Resin and Coatings Preparation by Cationic and Radical Photocrosslinking

**DOI:** 10.3390/molecules26247663

**Published:** 2021-12-17

**Authors:** Paulina Bednarczyk, Izabela Irska, Konrad Gziut, Paula Ossowicz-Rupniewska

**Affiliations:** 1 Department of Chemical Organic Technology and Polymeric Materials, Faculty of Chemical Technology and Engineering, West Pomeranian University of Technology, Piastów Ave. 42, 71-065 Szczecin, Poland; konrad.gziut@zut.edu.pl (K.G.); Paula.Ossowicz@zut.edu.pl (P.O.-R.); 2 Department of Materials Technology, Faculty of Mechanical Engineering and Mechatronics, West Pomeranian University of Technology, Piastów 19 Avenue, 70-310 Szczecin, Poland; izabela.irska@zut.edu.pl

**Keywords:** epoxy methacrylates, coatings, photopolymerization

## Abstract

This work involves the synthesis of hybrid oligomers based on the epoxy methacrylate resin. The EA resin was obtained by the modification of industrial-grade bisphenol A-based epoxy resin and methacrylic acid has been synthesized in order to develop multifunctional resins comprising both epoxide group and reactive, terminal unsaturation. Owing to the presence of both epoxy and double carbon–carbon pendant groups, the reaction product exhibits photocrosslinking via two distinct mechanisms: (i) cationic ring-opening polymerization and (ii) free radical polymerization. Monitoring of EA synthesis reactions over time using PAVs, MAAC and NV parameters, and the FT-IR method reveals that esterification reactions proceed faster at the start, exhibiting over 40% of conversion within the initial 60 min, which can be associated with a relatively high concentration of reactive sites and low viscosity of the reaction mixture at the initial reaction stage. With the further increase in the reaction time, the reaction rate tends to decrease. The control of the EA synthesis process can guide how to adjust reactions to obtain EAs with desired characteristics. Based on obtained values, one can state that the optimum synthesis time of about 4–5 h should be adopted to prepare EAs having both epoxy groups and unsaturated double bonds. The structure of the obtained EA was confirmed by FT-IR and NMR methods, as well as the determination of partial acid value and epoxy equivalent. Samples at various stages of synthesis were cured with UV radiation in order to study the kinetics of the process according to cationic and radical polymerization determined via photo-differential scanning calorimetry (photo-DSC) and real-time infrared spectroscopy (RT-IR) and then the properties of the cured coatings were tested. It turned out that the cationic polymerization was slower with a lower conversion of the photoreactive groups, as compared to the radical polymerization. All the obtained EA coatings were characterized by good properties of cured coatings and can be successfully used in the coating-forming sector.

## 1. Introduction

Epoxy acrylate resins are an important class of unsaturated compounds commonly known as vinyl ester resins (VERs) [1,2,3]. The latter was introduced almost simultaneously by Shell Chemical Company (Epocryl^®^ resins) and Dow Chemical Company (Derakane^®^ resins) in the mid-1960s as high-performance resins for engineering applications [1,2]. Vinyl ester resins are the product of the addition reaction of α-β unsaturated carboxylic acids to epoxy resins. Basically, the VEs formulations, thus the final polymer properties, can be tuned by a choice of: (i) the type of backbone component; (ii) the molecular weight of the resin; and (iii) the type and content of reactive diluent (if one is used) [3,4]. Most commercially-available VERs are based on diglicydyl ether of bisphenol A (DGEBA) or bisphenol A diepoxy resins of various molecular weights.

Furthermore, some special-purpose resins, that is, heat-resistant or flame-retardant vinyl esters, have been developed using epoxy phenol novolac resins and epoxy resins based on tetrabromobisphenol A, respectively. In conventional, industrially significant systems, monocarboxylic acids, such as acrylic (AA) and methacrylic acid (MAA), serve as a source of reactive unsaturation on the VERs end-group [1,3]. Moreover, crotonic and cinnamic acids have also been proposed for this reaction [5]. Vinyl ester resins can be used in a neat form or as a formulation containing a reactive diluent (i.e., monofunctional vinyl precursor -comonomer). Among available reactive diluents, styrene is the most preferred and widely used to date [6,7]. In principle, di(meth)acrylates can be cured via peroxides catalyzed free-radical mechanism (thermal- or redox-activated) or photopolymerization reactions [8]. However, it should be emphasized that the latter method is in favor, owing to low energy consumption, high speed of the process, and solvent-free formulations [9]. The commercial success of VERs is owed to the combination of the advantages of epoxy resins, such as excellent mechanical and thermal properties along with fast curing of unsaturated polyester resins [2,8]. Due to their excellent adhesive properties, high mechanical performance and outstanding chemical resistance, the resins in question play a significant role in various industrial applications. For example, VERs are widely employed as a matrix for high-performance fiberglass reinforced composites or as a main component of UV-curable coatings and ink formulations [2,8,10] as well as used in selective removal of pollutants, for example, non-transition metal ions or alkaline earth metal ions [11,12,13,14].

To date, only a few studies have been carried out to develop multifunctional resins comprising both epoxide group and reactive, terminal unsaturation (monoacrylate-terminated epoxy acrylate) [10,15,16,17]. Among them, growing interest has been paid to difunctional compounds synthesized from DGEBA/bisphenol A diepoxy resins and AA. The kinetics of carboxyl-epoxide addition esterification in the DGEBA-AA system have been studied in detail by Su Y. et al. They assumed that the reaction follows second-order kinetics with an activation energy of 63.9 kJ/mol [16].

It should be emphasized that the majority of difunctional EAs were designed to combine the functionalities of both UV- and thermal-curing. In this context, Park Y. et al. investigated the dual curing behavior of composition based on partially acylated DGEBA epoxy oligomers with varying content of a latent curing agent [10]. Su Y. et al. applied the same synthesis route to synthesize dual-curable difunctional epoxy acrylate with potential application as an adhesive sealant for liquid-crystal display (LCD) production [16]. More recently, Yildiz Z et al. synthesized bisphenol-A-based acrylated epoxy oligomers and employed them to improve the adhesion strength between polyester cords and rubber [15]. They found that the hydroxyl groups formed in the reaction between epoxy and acid can improve wettability and help the material to adhere to difficult surfaces. In fact, with increasing acrylate functionality, the resin polarity increases, resulting in an enhanced adhesion at the rubber–cord interface. In turn, Kardar P. et al. [17] utilized difunctional epoxy acrylate oligomers in UV curable coating compositions. The EA formulations, aside from resin, also contain multifunctional acrylate monomers as reactive diluents and alumina nano-particles (Al_2_O_3_ NPs).

The very first commercial multifunctional low viscosity resins were launched by Hexion Inc. under the trade name Epon. The most recognized product in their offer, coded as Epon 8111, is a combination of bisphenol A-based epoxy resin and acrylate [18]. According to the manufacturer, the latter can be employed in rapid set adhesives, patching compounds, and wear-resistant coatings. In our previous study [19], a series of novel trifunctional epoxy (meth)acrylate resins (EAs) containing at least one epoxy group and at least one acrylate group were synthesized via the addition of (meth)acrylic acid to the triglycidyl ether of trimethylolethane (TMETGE). In addition, the photocurable compositions based on synthesized EAs with cationic and radical photoinitiator were investigated in terms of UV-curing kinetics and coatings performance.

In this work, the modification of epoxy resin with methacrylic acid, allowing to obtain a polymer containing both acrylic and epoxy groups, has been reported. At first, optimization of modification procedure was followed by a kinetics study, where molar ratio, catalyst dosage and process temperature remained constant and set to optimal conditions, whilst the synthesis time was varied from 60 to 300 min. Pre-polymers properties were studied regarding their chemical structure (FT-IR studies), non-volatile-matter content (NV), partial acid value (PAVs), epoxide equivalent (EE) and viscosity. Moreover, substrates and synthesized epoxy methacrylates were investigated by nuclear magnetic resonance spectroscopy (^1^H NMR). Subsequently, photoreactive coating compositions were prepared from the obtained methacrylated epoxides and cured using UV radiation. The prepolymers collected during the reaction were subjected to cationic photopolymerization, while the final product was tested for cationic and radical photopolymerization. The samples were tested for the kinetics of the process and the properties of the cured coatings.

## 2. Results and Discussion

### 2.1. Synthesis Parameters and Properties of Obtained Epoxy Methacrylate Resin

**Figure 1:**
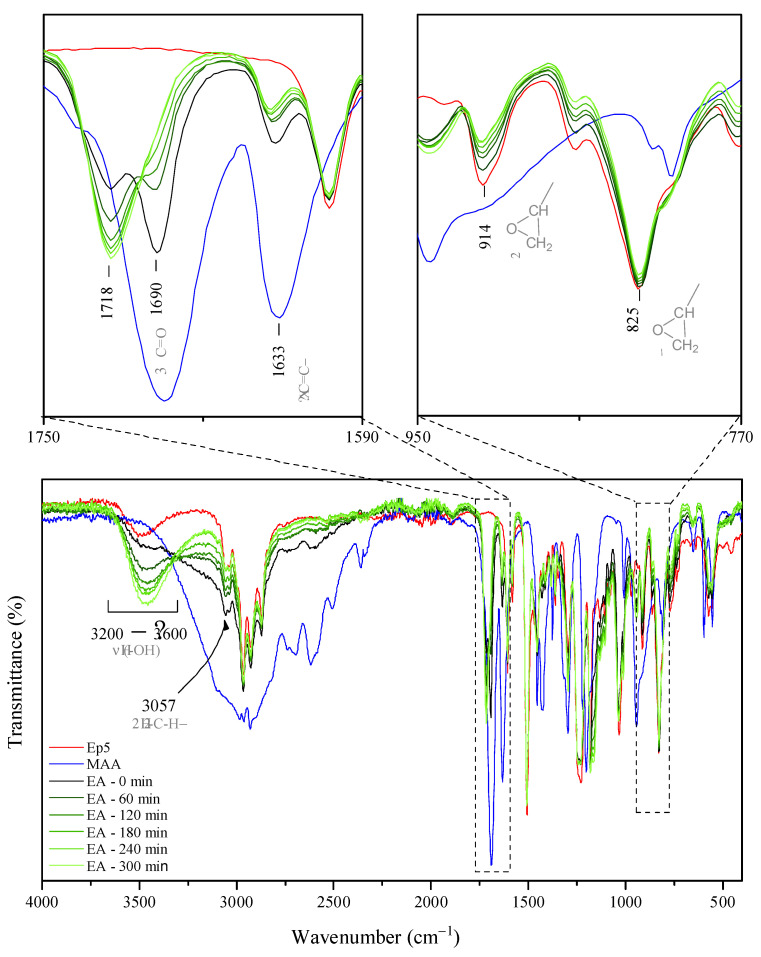
FT-IR spectra of epoxy resin, methacrylic acid, and EA prepolymers collected throughout the reaction time (the digit at the end of sample code indicates the reaction time; “0” stands for reaction mixture preheated to 70 °C). Reported spectra were recorded at room temperature, as an average of sixteen scans.

Several factors, such as MAA to epoxy resin feed ratio, amount of catalyst, process temperature, and finally, reaction time, have influenced the progress of addition reaction of the MAA to the epoxy resin. The synthesis parameters were selected based on a literature survey [10,20,21,22,23] and several preliminary runs carried out at different reaction temperatures and catalyst doses. The best temperature turned out to be 90 °C, and the catalyst dose of 0.8 wt% was assumed as the optimal amount. However, employing higher temperatures or an increased amount of catalyst appears to accelerate the esterification reactions considerably, making the process hard to control. That is to say, partially gelled, amber-like products were obtained. A possible explanation of this phenomenon may be found in the chemical structure of resin, which is quite complex, comprising hydroxyl groups originating from epoxy resin and formed during the acrylation (see Figure 1). In turn, the latter can randomly react with remaining epoxy groups of epoxy resin, leading to the crosslinking of polymer chains [24].

As far as reaction time is concerned, the model synthesis of the epoxy methacrylate (EA) system was performed and characterized in detail in order to establish the impact of the extent of the reaction on the chemical structure, conversion of the substrate, and finally, functional properties of epoxy methacrylate pre-polymers. The samples were collected every hour for a total of 5 h and the progress of the reaction was monitored by FT-IR spectroscopy. The absorption bands corresponding to the vibrations of carboxyl and epoxy groups (middle and right panel in Figure 1, respectively) are precious in assessing the evaluation of the reaction progress in the investigated system. As the reaction progresses, from Figure 1 it can be observed that the intensity of the IR absorption bands arising from -C-H (~3057 cm^−1^), C-O (~914 cm^−1^) and C-O-C (~825 cm^−1^) stretching vibrations of oxirane groups [25,26,27] gradually decrease, thus confirming that the ring-opening of epoxy groups took place. Moreover, one can note that the peak at 1690 cm^−1^ originating from the carbonyl stretching mode in MAA [28,29] (see the zoomed view of the spectra in the top right panel) becomes less intense and finally indistinguishable (after 5 h of mixing). Whereas an additional absorption peak corresponding to C=O stretching vibrations develops at 1718 cm^−1^, thereby suggesting that new bonds occur due to the reaction of carboxyl groups of MAA and epoxide groups of epoxy resin, that is, ester bonds. This is in agreement with the previous studies reporting the shift of the C=O band after the esterification reaction [30]. The progress of ring-opening reactions between epoxide groups and MAA is also highlighted by a gradual increase in the intensity of broadband occurring in the range of 3600–3200 cm^−1^, due to -O-H groups’ evolution [27,31,32] resulting from the epoxide ring-opening.

**Figure 2:**
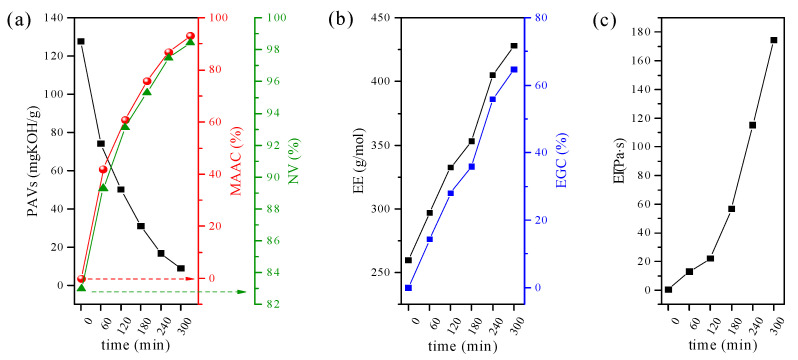
(**a**) The partial acid value (PAVs) vs. methacrylic acid conversion (MAAC) and non-volatile-matter content (NV); (**b**) epoxy equivalent (EE) vs. epoxy group conversion (EGC) and (**c**) viscosity (η) as a function of reaction time.

For better understanding, the product evolution over the reaction time, the non-volatile-matter content (NV), partial acid value (PAVs) and epoxy equivalent (EE) were also tracked. Each of these can be regarded as a measure of reaction progress. Thus, in the present study, the relationship between process time and the % of reacted epoxide groups as well as % of reacted MAA was established and expressed as epoxy group conversion (EGC) and methacrylic acid conversion (MAAC), respectively (Figure 2). Generally, as the reaction time proceeds, an increase in both MACC (Figure 2a) and EGC (Figure 2b) can be seen, which supports the high efficiency of reactions occurring in the system. A close inspection of PAVs, MAAC, and NV (originating mostly from unreacted portions of MAA) reveals that esterification reactions proceed faster at the start, exhibiting over 40% of conversion within the initial 60 min. With the further increase in the reaction time, the reaction rate continues to fall, yielding only 5% of MAA conversion in the final phase. The observed trend can be explained by considering that at the initial reaction stage, the concentration of reactive sites is relatively high; thus, the possibility of reaction between the acid and epoxide groups is high, while further reduction of reactive sites impedes the process [20]. Moreover, it is worth noting that the viscosity of the resin increases substantially with the reaction time (Figure 2c), which also may alter the overall reaction rate. That is to say, systems based on Ep5 and MAA with 50% conversion of epoxy groups are characterized by higher viscosity values when compared to analogous systems based on Ep6 and acrylic acid [33].

The above results are of the utmost importance because they can guide how to adjust reactions to obtain EAs with desired characteristics. Based on obtained values, one can state that the optimum synthesis time of about 4 h should be adopted to prepare EAs that have both epoxy groups and unsaturated double bonds. In the EA, the epoxy conversion at 240 min is close to 50%, while MAA conversion reaches 89%. Prolonging the reaction time leads to a further increase in the epoxy group conversion up to 52% in 5 h, reducing the number of functional groups capable of cationic polymerization at the same time. 

**Figure 3:**
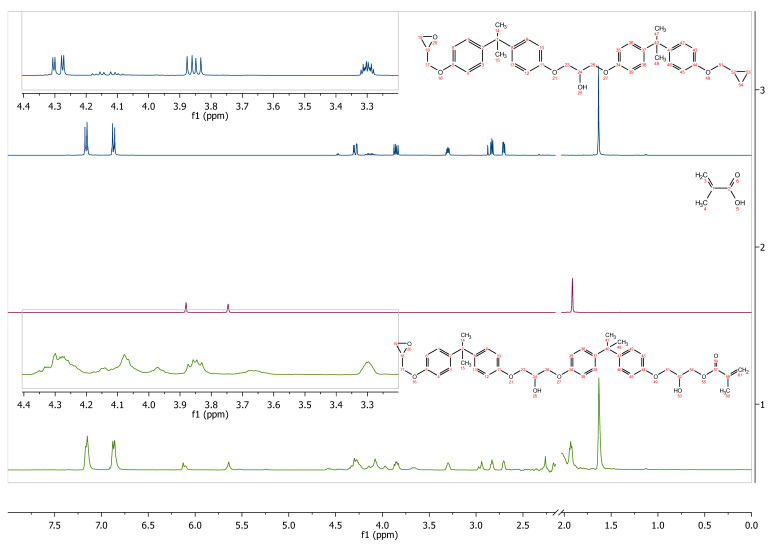
^1^H NMR spectrum (d-acetone) of epoxy resin, methacrylic acid, and EA prepolymer.

The ^1^H NMR spectra also confirm the structure of the obtained epoxy methacrylate. Figure 3 shows the ^1^H NMR spectra of the dianate epoxy resin, methacrylic acid, and prepared epoxy methacrylate. In both Ep5 and EA spectra, signals about 7.1–7.2 ppm and 6.8–6.9 ppm were observed, attributed to the resonance of aromatic protons, respectively. The signal at 1.63 ppm corresponds to the resonance of methyl protons between the phenyl rings, while the group of signals in the region 2.6 and 3.1–3.3 and 3.8–4.2 corresponds to glycidol protons. In addition, the methacrylic acid and product spectra show signals from the vinyl group at 6.10/5.64, and 6.21/5.84, respectively.

Further evidence from the ^13^C NMR spectrum shows that carbon resonance accords well with the predicted molecular structure (Supplementary Materials). 

Structurally, for the obtained compound, the signals related to the epoxy ring emerge no disappear, whereas the new ones due to allyl groups appear (Supplementary Materials Figure S1). Thus, the integration of signals from protons in the double bond agrees with the integration of signals from epoxides.

This result confirms that the proposed structure contain both methacrylate and epoxy moieties. 

**EA-^1^H NMR** (400 MHz, Acetone-d6): δ in ppm 7.16 (d, J = 8.4 Hz, 8H), 6.87 (d, J = 8.8 Hz, 8H), 6.12 (d, J = 8.5 Hz, 1H), 5.64 (s, br, 1H), 4.26–4.30 (m, 4H), 4.07 (d, J = 5.2 Hz, 4H), 3.97 (m, 1H), 3.85 (dd, J = 11.0, 6.3 Hz, 2H), 3.60–3.71 (m, 1H), 3.34–3.25 (m, 1H), 2.94 (s, 1H), 2.83 (t, J = 4.7 Hz, 1H), 2.71 (dd, J = 5.2, 2.6 Hz, 1H), 2.27 (s, 2H), 1.92 (d, J = 5.7 Hz, 2H), 1.63 (s, 12H); ^13^C NMR (100 MHz, Acetone-d6) δ in ppm: 166.58, 156.63, 143.32, 136.43, 127.63, 127.61, 127.56, 125.03, 113.90, 69.16, 67.79, 67.68, 65.72, 49.77, 43.56, 41.37, 30.51, 29.76, 17.57.

### 2.2. The Photocuring of the Epoxy Methacrylate Pre-Polymers

**Figure 4:**
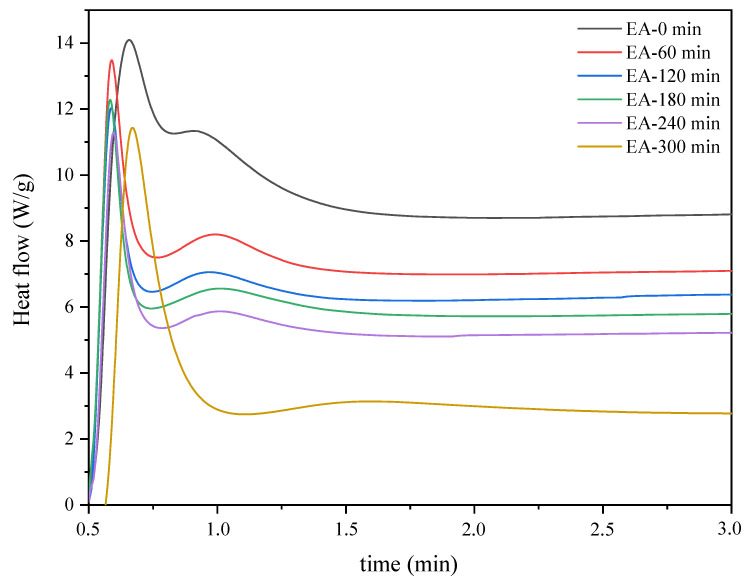
Photo-DSC thermograms corresponding to the photocuring of the reaction mixtures obtained at one-hour intervals (photo-DSC method; mercury UV lamp, 280–480 nm, 100 mW/cm^2^; composition of the photoreactive formulations: 96 wt% of EA prepolymer, 4 wt% of cationic PI).

In a first approach, the photoinitiated cationic polymerization of the pre-polymers collected throughout the reaction was monitored through the photo-DSC method. Photo-DSC offers a simple method of characterizing the UV-curing kinetics for the photopolymerization of UV-cured materials. Therefore, the profiles for the heat of reaction versus time provided by photo-DSC can be used to describe the photoinduced reaction kinetics and evaluate the polymerization rate. The compositions used in the study included reaction mixtures obtained at one-hour intervals and a cationic photoinitiator (4 wt%). Figure 4 shows the photocalorimetric exotherms for the photoinitiated cationic polymerization of the prepolymers collected throughout the reaction. As explained earlier, the tested samples are mixtures of epoxy methacrylate oligomers with unreacted epoxy resin and methacrylic acid, as well as fully acrylated epoxy methacrylate oligomers. As the reaction proceeds, a mixture with more reacted epoxy groups, and methacrylic acid is obtained. Therefore, the various reaction mixtures have different curing rates. 

In the plot, the UV irradiation was initiated the reaction mixtures with a cationic photoinitiator, and the photopolymerization reaction occurred immediately in the presence of UV light. The total area under the thermogram curves was the highest for samples with lower viscosity (less than 20 Pa·s). It decreased with the decrease in the concentration of epoxy groups in the reaction mixture. This phenomenon can be reasonably ascribed to the epoxy methacrylate oligomers’ increased molecular weight and conversion degree. As the reaction proceeded, the viscosity of the samples increased significantly, resulting in a decrease in reactivity. Thus, the heat flow decreased due to a decrease in the relative abundance of reactive epoxy groups and viscosity, resulting in a slower reaction rate. In the initial stage of the EA synthesis reaction, there are many unreacted epoxy groups and MAA molecules, hence a double peak is observed, which probably corresponds to the photopolymerization of the epoxy groups of the obtained EA prepolymer as well as unreacted epoxy resin. As the reaction proceeds, the peak corresponding to the photopolymerization of the epoxy groups of the unreacted epoxy resin disappears. As a consequence, a distinct exothermic peak is obtained which corresponds to the actual photopolymerization reaction of the obtained epoxy methacrylate prepolymer.

As the reaction progresses and the epoxy groups in the reaction mixture decrease, the tack-free time is extended from 45 to 90 s (Table 1). Thus, a 50% reaction of the epoxy groups (EA-240 min) resulted in almost a twofold extension of tack-free time. The hardness of the cured coatings correlated with the conversion of the crosslinked polymers. Thus, the reduction of the polymerization rate due to the reduction of epoxy groups in the samples also results in a reduction in the hardness of the cured coatings, as well as a reduction in scratchability. However, the gloss of the cured coatings increased, and there was no significant change in yellowness.

### 2.3. The Cationic and Radical Photopolymerization of Epoxy Methacrylates

**Figure 5:**
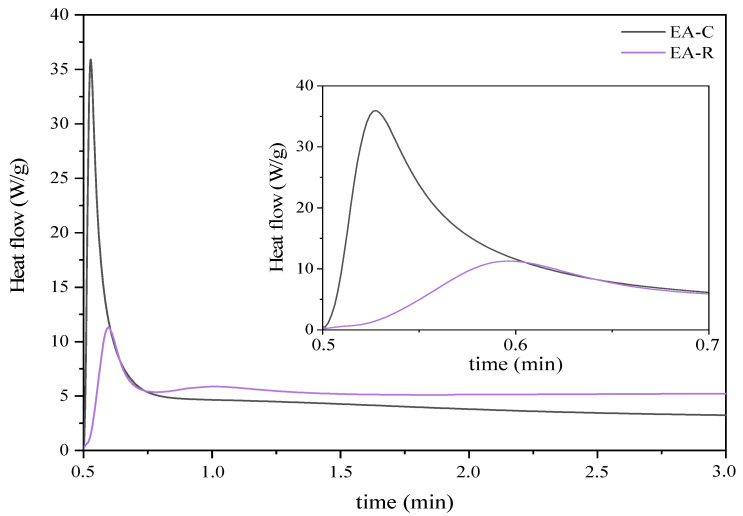
Photo-DSC thermograms corresponding to the cationic (EA-C) and radical (EA-R) photocuring behavior (photo-DSC method; mercury UV lamp, 280–480 nm, 100 mW/cm^2^; composition of the photoreactive formulations: 96 wt% of EA prepolymer, 4 wt% of cationic or radical PI).

Moreover, it is worth emphasizing that, owing to the presence of both epoxy and double carbon–carbon bonds in the polymer chain; the resulting EAs are expected to exhibit curing behavior via two distinct mechanisms: (i) cationic ring-opening polymerization; and (ii) free radical polymerization [34]. Therefore, a pre-polymer with an epoxy conversion degree of 50% (EA-240 min) was selected to observe the cationic and radical photopolymerization behavior. Figure 5 shows the photo-DSC exotherms for the photopolymerization of the investigated UV-curable systems. It was observed that radical photopolymerization of EA prepolymer exhibits higher exotherms and much faster polymerization reactivity than for the cationic process, which polymerizes much slower. In addition, unlike the cationic process, only one exothermic peak is observed in the case of the radical process, which probably indicates the polymerization of only methacrylate groups attached to the prepolymer chain.

**Figure 6:**
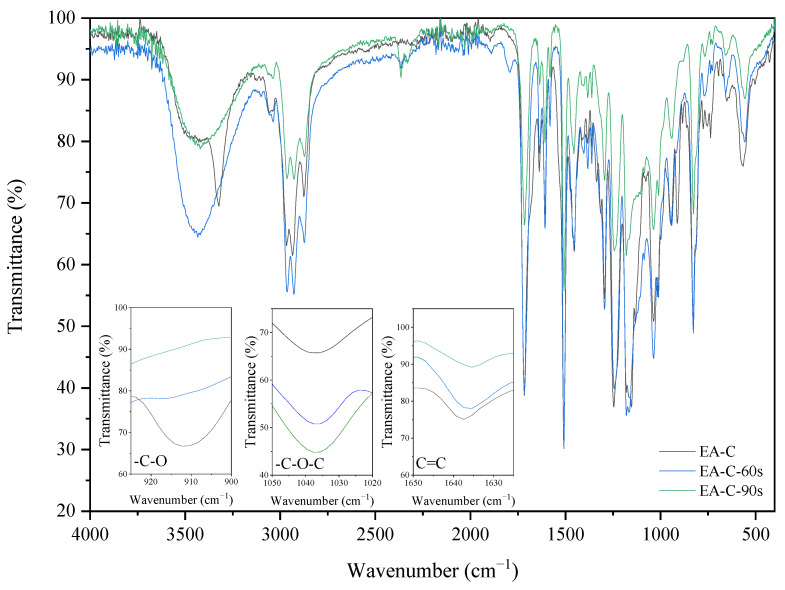
The FT-IR spectra of the EA-C sample before and after 60 and 90 s of UV irradiation (composition of the photoreactive formulations: 96 wt% of EA prepolymer, 4 wt% of cationic PI).

**Figure 7:**
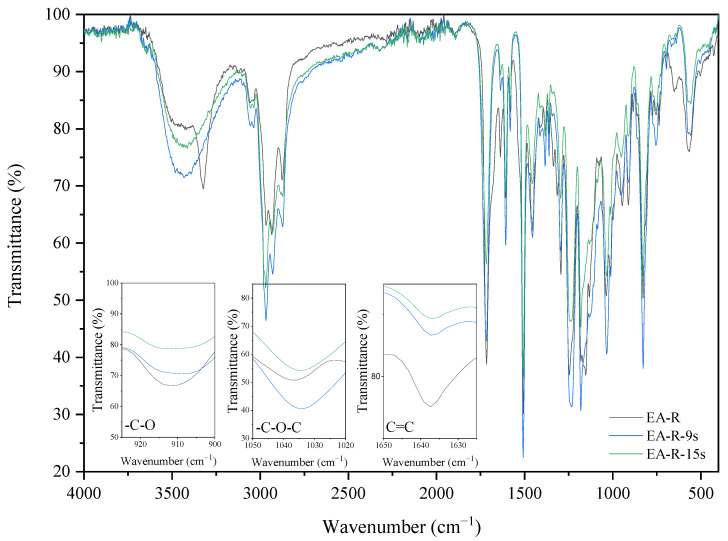
The FT-IR spectra of the EA-R sample before and after 9 and 15 s of UV irradiation (composition of the photoreactive formulations: 96 wt% of EA prepolymer, 4 wt% of radical PI).

IR spectra analyses also observed the cationic and radical crosslinking polymerization of the EA prepolymer. The two investigated coating compositions (with cationic-EA-C and radical photoinitiators–EA-R) exhibit different IR spectra after the curing process. The spectra differences of uncured and cured EA samples can be observed by revealing a presence of characteristic absorption bands for epoxy ring stretching vibration (-C-O), ether groups (-C-O-C), and double bonds derived from the methacrylic groups (C=C). The presence of epoxy groups in IR spectra was proved from the presence of strong bands at 915 cm^−1^ (γ_C-O_ epoxy), the ether groups at 1036 cm^−1^, and double bonds at 1635 cm^−1^ (γ_C=C_ methacrylate double bond). Figure 6 and Figure 7 show the IR spectra recorded before and after various exposure times, up to 90 s, for EA samples with cationic (C) or radical (R) photoinitiator (Deuteron 1240 or Omnirad TPOL (4 wt%)), respectively. As expected, the IR spectra at 915 cm^−1^ exhibit a characteristic absorption band for epoxy groups, the intensity of which gradually decreases with the extension of cationic ring-opening polymerization. Simultaneously, one can observe the increase of characteristic absorption bands for ethers (-C-O-C) at 1036 cm^−1^ and the presence of methacrylic double bonds at 1635 cm^−1^. The crosslinking of radical polymerization of EA-R composition was investigated by analyzing the same peaks, in particular the double bonds’ characteristic peak at 1635 cm^−1^. As the curing time proceeds, the latter diminishes in intensity, suggesting polymerization by unsaturated bonds derived from the methacrylate double bonds. In this case, the presence of epoxy groups is also confirmed after the curing process.

**Figure 8:**
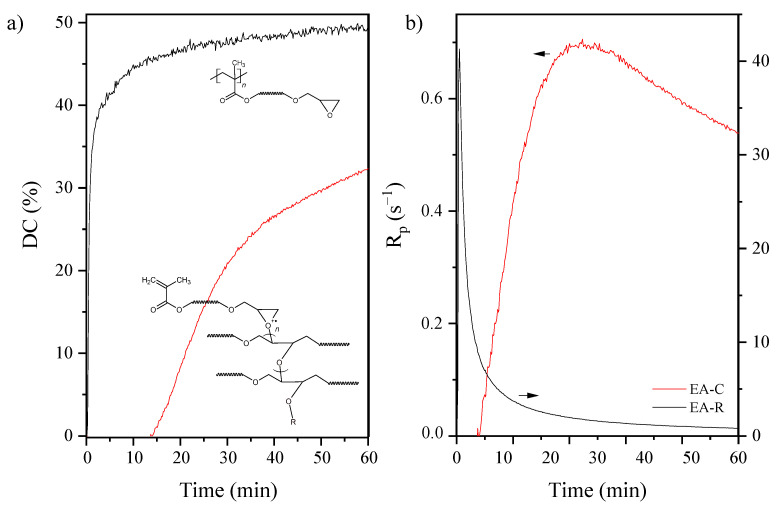
The epoxy and unsaturated double bond conversion (**a**) and the photopolymerization rate (**b**) curves of the EA under the different mechanisms of polymerization: C–cationic or R–radical (RT-IR method; mercury UV lamp, 280–400 nm, 10 mW/cm^2^; composition of the photoreactive formulations: 96 wt% of EA prepolymer, 4 wt% of cationic or radical PI).

Kinetics of photopolymerization is useful for understanding both curing rate and curing degree, particularly in the context of influencing the cationic or radical mechanism of the photopolymerization process. The epoxide and unsaturated double bond conversion (DC), as well as photopolymerization rate (Rp) versus irradiation time, were investigated employing the FT-IR method (Figure 8). As expected, the progressive disappearance of the various IR bands characteristic of the epoxy or methacrylate double bonds was observed. The peak areas determined the extent of epoxy groups’ reaction at 915 cm^−1^ due to C-O stretching in the epoxy ring. The peak of the epoxy group at 915 cm^−1^ decreases during the UV curing process, as shown in the IR spectra. Simultaneously, the extent of double bond reactions of EA-R was determined by the peak areas of the double bond peak at 1635 cm^−1^.

The cationic photopolymerization proceeds slowly, probably due to severe mobility restrictions in the solid uncured film and reaches only 32 % conversion after 60 min UV exposure. From the initial slope of the recorded polymerization profile, the formulation reactivity was evaluated as 0.7 s^−1^ (reactivity understood as the loss of epoxide groups over time). Other results have been obtained by using a radical photoinitiator. In this case, the double bond conversion was about 50 % after the same time as the cationic process, and the polymerization rate was found to be 40 s^−1^. It is well-known that during the reaction of the UV-curing system, the polymer’s mobility and the diffusion capacity of the active radicals greatly influence the conversion efficiency [35]. Therefore, the high rate of radical photopolymerization allowed for the increased conversion of unsaturated bonds. In turn, in the case of cationic photopolymerization, the process was carried out at a low speed. Thus, a lower conversion of epoxy groups was obtained. In addition, as the reaction proceeded, the viscosity of the system increased. In high-viscosity systems, the diffusion of cations during the progress of the reaction was limited, leading to a reduction in the conversion of epoxide ring-opening photopolymerization.

Table 2 shows the basic properties of the cured coatings obtained by the cationic and radical process with various times of exposure to UV radiation. Conventionally, the cure extent of a coating can also be followed through pendulum hardness measurements (Persoz), which are correlated with the conversion of the crosslinked polymer. The Persoz method consists of monitoring the damping time of the oscillations of a pendulum placed onto the polymer sample coated on a glass plate. Persoz values typically range between 50 s for soft elastomeric materials and 350 s for hard and glassy polymers, to reach a maximum value of 400 s for mineral glass [36]. The coating hardness varies with the curing time. The following trends were observed: (i) the cationic ring-opening photopolymerization leads to similar hardness as radical methacrylate double bond photopolymerization, but in a long time; (ii) 90 s is sufficient to obtain a glossy coating with high hardness in the case of the cationic process, while in the case of the radical process, a similar hardness was obtained after 15 s of exposure to UV radiation, that is, in a six times shorter amount of time. Nevertheless, both methods allow the obtaining of cured coatings with satisfactory properties and could be practicable in an industrial application.

## 3. Materials and Methods

### 3.1. Materials

The epoxy resin (Epidian 5^®^ (Ep5); with epoxide number of 0.48–0.51 mol/100g and viscosity ranging from 20,000 to 30,000 mPa·s at 25 °C) were purchased from “Organika-Sarzyna” S.A., Nowa Sarzyna, Poland. Hence, the following materials were used to synthesize hybrid oligomer: methacrylic acid (MAA), stabilized, with the purity of 99.5%, was supplied by Acros Organics, Geel, Belgium. Triphenylphosphine (PPh_3_), Apollo Scientific, Bredbury, UK, was used as a catalyst in the reaction between Ep and MAA, while hydroquinone (HQ, Acros Organics, Geel, Belgium) was used as a polymerization inhibitor. All chemicals were employed as received. 

The following titration reagents and indicators were used: glacial acetic acid, toluene, potassium hydroxide standard solution 0.1 M in ethanol (KOH) and crystal violet purchased from Chempur (Piekary Slaskie, Poland); chloroform form P.P.H. Stanlab (Lublin, Poland); ethyl alcohol from Avantor (Gliwice, Poland), tetraethylammonium bromide provided by Acros Organics (Geel, Belgium), perchloric acid standard solution 0.1 M in glacial acetic acid supplied by Fischer Chemicals (Zurich, Switzerland), Phenolophtalein 1% in ethyl alcohol solution from Eurochem BGD (Tarnów, Poland). All chemicals were analytical grade and were used as received. 

Bis(dodecylphenyl)iodoniumhexaflouroantimonate in propylene carbonate (Deuteron UV 1240, Deuteron) was applied as a cationic photoinitiator and ethyl(2,4,6-trimethylbenzoyl)-phenyl phosphinate (Omnirad TPOL, IGM Resins) was applied as a radical photoinitiator. In addition, glycidyl methacrylate was used as a reactive diluent.

### 3.2. Synthesis of Hybrid Oligomer Based on the Epoxy Methacrylate Resin

**Figure 9:**
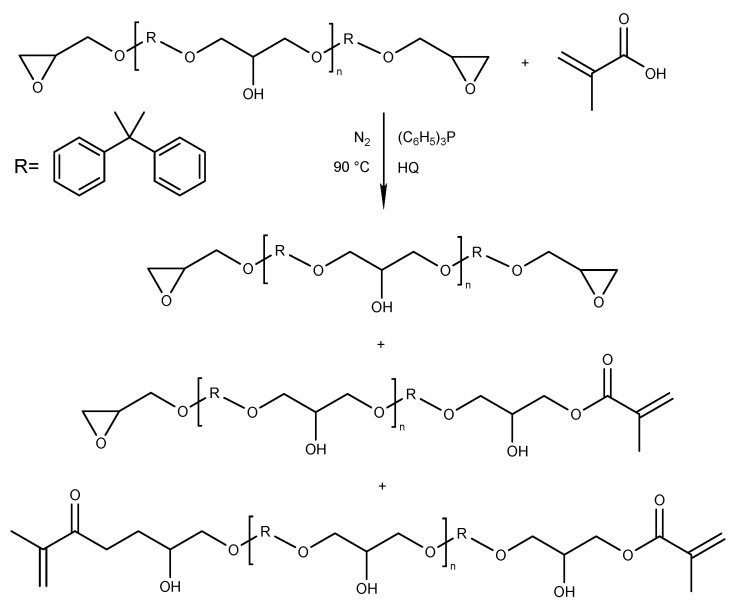
Synthesis of epoxy methacrylate resin.

The synthesis of epoxy methacrylate (EA) resin was carried out in a 250 mL three-neck glass reactor (equipped with a thermometer, a condenser, a nitrogen inlet, and a mechanical stirrer), into which bisphenol A type epoxy resin and hydroquinone as a radical scavenger (0.0075 wt% based on total batch weight) was added into the reactor at room temperature. Then, methacrylic acid (0.5 mol relative to resin epoxy value) and catalyst–triphenylphosphine in the amount of 0.8 wt% (relative to the mass of MAA) were added. The mixture was heated to 70 °C using an oil bath and stirred (120 RPM) to obtain the homogenous mixture. The final reaction temperature was set to 90 °C, and the process was conducted for 5 h in a nitrogen atmosphere, with stirring. The above synthesis parameters were established based on a literature survey [10,20,21,22,23] and our own experience [19,36]. The reaction products appear as colorless, transparent, viscous liquids. The general reaction scheme for the addition of methacrylic acid to epoxy resin, together with the expected products, is presented in Figure 9.

### 3.3. Characterization and Performance Evaluation

The infrared spectra were acquired with a Thermo Nicolet 380 FT-IR spectrometer. Sixteen scans were averaged for each sample in the range of 4000–400 cm^−1^_,_ at room temperature.

The prepared epoxy methacrylates were identified by ^1^H NMR and ^13^C NMR. The ^1^H and ^13^C NMR spectra were recorded in d_6_-acetone containing 0.03% (*v/v*) TMS (tetramethylsilane) on a BRUKER DPX-400 Avance III HD spectrometer (Billerica, MA, USA) operating at 400.13 MHz (^1^H) and 100.62 MHz (^13^C).

The non-volatile-matter content (NV) was evaluated thermogravimetrically, using moisture analyzer MAX 60/NP (Radwag, Poland), according to ISO 3251:2019 standard. The analysis was performed at 140 °C for 30 min. The sample weight was approximately 1 g. NV (%) = (m_2_/m_1_) × 100%, where m_1_ is the weight of the EA sample; m_2_ is the residual weight of the sample after heating.

Partial acid values (PAVs) were determined by colorimetric titration according to EN ISO 2114:2000 standard, following the procedure described in detail elsewhere [19]. 

The PAVs values were used to estimate methacrylic acid conversion (MAAC) according to the following equation: $MAAC=100-\frac{PAV_{s}\cdot 100}{PAV_{s0}},$ where PAV_s0_ is the initial value of PAV_s_ (mg KOH/g).

Epoxy equivalent (EE) was determined by means of colorimetric titration, according to EN ISO 3001:1999 standard following the procedure reported in our previous work [19]. 

Epoxy group conversion (EGC) was estimated from EE measurements via the following formula: $EGC=100-\frac{EE_{0}*100}{EE_{measured}}\left[\%\right],$ where: EE_0_ is the initial value of EE (g/mol); EE_measured_–EE value at a certain time.

The viscosity tests were carried out using a cone-plate viscometer LAMY RM-100 plus CP 2000 at room temperature.

### 3.4. Preparation of Coating Compositions and Cured Films

The coating compositions have been formulated using synthesized epoxy methacrylates and 4 wt% PI (Deuteron UV 1240 in cationic process and Omnirad TPOL in radical process). The components were stirred together under dark conditions until a homogeneous mixture was obtained. Subsequently, the curing solution was applied to the glass substrates employing a gap applicator (120 µm). The polymeric film was cured under a light source (UV lamp, Aktiprint-mini 18-2, type: UN50029, Technigraf GmbH) at room temperature and irradiated under UV light with an intensity of 200 mW/cm^2^ to dryness.

### 3.5. Characteristics of the Photopolymerization Process and Properties of Cured Coatings

The UV-curing process of epoxy methacrylate varnishes was isothermally monitored (25 °C) for 15 min by means of photo-DSC apparatus (Q100, TA Instruments, New Castle, DE, USA) equipped with UV light emitter Omnicure S2000 (280–480 nm, 100 mW/cm^2^, Excelitas Technologies, Mississauga, ON, Canada). As a result, a polymerization solution was composed of epoxy methacrylate resin and 4 wt% of photoinitiator (Deuteron UV 1240 in cationic process and Omnirad TPOL in radical process).

Fourier transform infrared spectra (FT-IR) were obtained on a Nicolet iS5 instrument. The resolution is 4 cm^−1^, and the scanning range is 400–4000 cm^−1^. The recording interval of the spectrum was 10 s. Series real-time IR (RT-IR) was used to determine the conversion of epoxide groups of methacrylic double bonds. More importantly, this spectroscopic technique permits in situ monitoring of the chemical processes via mimicking the disappearance of the characteristic bands of the reactive monomer subjected to UV exposure [37]. The EA and an initiator mixture were placed in a mold made from glass slides and spacers of 15 mm in diameter and 1.2 in thickness. The samples were placed in the compartment of a Fourier transform infrared spectrometer and were simultaneously exposed to a UV light source (mercury UV lamp, 36 W, 280–400 nm, 10 mW/cm^2^) and an IR analysis light beam. The absorbance change of the epoxide group (C-O) and methacrylate double bond (C=C) peak area was correlated to the extent of polymerization. The degree of conversion (DC), can be expressed by the following relations: DC (%) = (A_0_-A_t_) · 100/A_0_, where A_0_ is the initial peak area before irradiation and A_t_ is the peak area at time t. The photopolymerization rate (Rp) was calculated by the following relations: Rp = d_DC_/d_t_, where t is the time of irradiation [35].

The following tests were performed in order to evaluate the properties of cured coatings: tack-free time, pendulum hardness test, gloss and yellowness index. Tack-free time was measured as a surface cure time according to ISO 9117. It is the time at which the coating is deemed to be properly adhered to and achieves the final technical parameters. The hardness of coatings was tested using Persoz pendulum hardness on the glass substrate (TQC Sheen, Capelle an den Ijssel, The Netherlands) according to ISO 1522 standard. Gloss was measured by spectrometer GLS at an angle of 20 degrees (SADT Development Technology Co. Ltd., Beijing, China) according to ASTM D523. Yellowness Index is a number calculated from spectrophotometric data that describes the change in the color of test samples. This parameter was measured according to ASTM E313 using precision colorimeter NH-145 (3NH Technology Co. Ltd., Shenzhen, China).

## 4. Conclusions

In this paper, the synthesis of epoxy methacrylate resin (EA) by modification of epoxy resin with methacrylic acid has been shown. Moreover, coatings preparation, based on synthesized resin and using UV radiation has been presented. The obtained EA prepolymer, which contains both epoxy and vinyl groups in their structure (confirmed by 1H NMR analysis), made it possible to produce coatings by two different photopolymerization mechanisms-cationic or radical. The optimization of the modification procedure, as well as the photo-crosslinking process, was followed by a kinetics study. The photoinitiated cationic polymerization of the prepolymers was monitored by means of the photo-DSC and FT-IR methods. Heat flow peaks related to the reaction kinetics of EAs prepolymers decreased as the reaction progresses, which is due to a decrease in the number of epoxy groups in the prepolymer chain at the expense of adding methacrylic groups. It also obtained cured coatings with a single exothermic peak, proving the photopolymerization of photoreactive groups in the obtained epoxy acrylate prepolymer. The properties of the cured coatings were characterized. Moreover, it has been shown that the radical process leads to similar properties of cured coatings as the cationic process does, which can be obtained in a shorter time.

## Figures and Tables

**Table 1:** The properties of the cured coatings (mercury UV lamp, 280–400 nm, 200 mW/cm^2^; composition of the photoreactive formulations: 96 wt% of EA prepolymer, 4 wt% of cationic PI).

Sample	Tack-Free Time (s)	Hardness (s)	Scratchability (N)	Gloss (GU)	Yellowness Index
EA-0 min	45	363 ± 2	2	160 ± 2	3.90 ± 0.2
EA-60 min	45	358 ± 2	2	168 ± 2	4.08 ± 0.3
EA-120 min	45	346 ± 2	2	169 ± 2	4.29 ± 0.3
EA-180 min	60	343 ± 1	1	190 ± 2	3.97 ± 0.2
EA-240 min	80	319 ± 1	1	197 ± 2	3.98 ± 0.2
EA-300 min	90	299 ± 1	1	197 ± 2	4.23 ± 0.2

**Table 2:** The basic properties of the cured coatings depend on the photopolymerization (C–cationic, R–radical) and the duration of UV radiation exposure time (mercury UV lamp, 280–400 nm, 200 mW/cm^2^; composition of the photoreactive formulations: 96 wt% of EA prepolymer, 4 wt% of cationic PI).

Sample	Tack-Free Time (s)	Curing Time (s)	Hardness (s)	Gloss (GU)	Yellowness Index
EA-C	60	30	139 ± 1	3 ± 2	3.19 ± 0.2
		45	260 ± 1	25 ± 2	3.29 ± 0.2
		60	268 ± 2	100 ± 2	3.48 ± 0.2
		75	276 ± 2	150 ± 2	3.70 ± 0.3
		90	296 ± 2	157 ± 2	3.71 ± 0.3
EA-R	9	3	140 ± 1	71 ± 2	3.18 ± 0.2
		6	161 ± 1	82 ± 2	3.70 ± 0.2
		9	175 ± 2	110 ± 2	3.99 ± 0.2
		12	282 ± 2	129 ± 2	4.07 ± 0.3
		15	299 ± 2	134 ± 2	4.21 ± 0.3

## Data Availability

Most of the data are provided in this work and in Supplementary Data. Other data that support the findings of this study are available from the corresponding author upon reasonable request.

## Supplementary Materials

The following are available online, Figure S1: ^13^C NMR spectra (d-acetone) of epoxy resin, methacrylic acid, and EA prepolymer.

## Sample Availability

Samples of the epoxy methacrylate resin are available from the authors.
