# Supplementary material for: Synthesis of Epoxy Methacrylate Resin and Coatings Preparation by Cationic and Radical Photocrosslinking
Source: Molecules. 2021 Dec 17;26(24):7663. doi: 10.3390/molecules26247663 (PMC8706006; doi:10.3390/molecules26247663)
Supplement: Supplementary file 1 [file molecules-26-07663-s001.zip › molecules-1481136-supplementary.pdf]

Supplementary Materials: Synthesis of epoxy methacrylate resin and coatings preparation by cationic and radical photo-crosslinking

Paulina Bednarczyk [1,*], Izabela Irska [2], Konrad Gziut [1] and Paula Ossowicz-Rupniewska [1]

[1] West Pomeranian University of Technology in Szczecin, Faculty of Chemical Technology and Engineering, Department of Chemical Organic Technology and Polymeric Materials, Piastów Ave. 42, 71-065 Szczecin, Poland

[2] West Pomeranian University of Technology in Szczecin, Faculty of Mechanical Engineering and Mechatronics, Department of Materials Technology, Piastów 19 Avenue, 70-310 Szczecin, Poland

* Correspondence: bednarczyk.pb@gmail.com or paulina.bednarczyk@zut.edu.pl

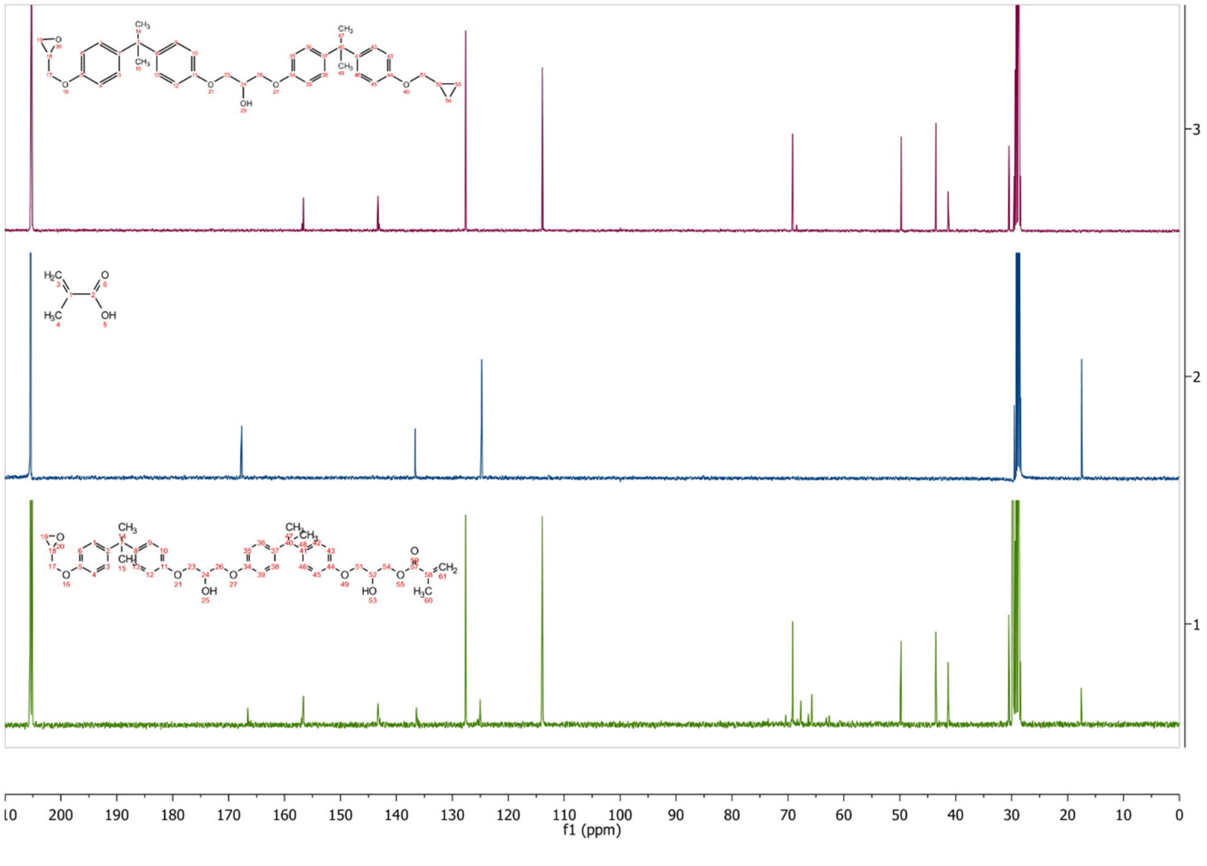

Figure S1. ^{13}C NMR spectra (d-acetone) of epoxy resin, methacrylic acid, and EA prepolymer.
